# Generation Z’s Shopping Behavior in Second-Hand Brick-and-Mortar Stores: Emotions, Gender Dynamics, and Environmental Awareness

**DOI:** 10.3390/bs15040413

**Published:** 2025-03-24

**Authors:** Veronika Harantová, Jaroslav Mazanec

**Affiliations:** Department of Quantitative Methods and Economic Informatics, Faculty of Operation and Economics of Transport and Communications, University of Zilina, 010 26 Zilina, Slovakia

**Keywords:** correspondence map, generation Z, consumer behavior, second-hand clothing, consumer attitudes

## Abstract

This study investigates the shopping behavior of Generation Z towards second-hand clothing in Slovakia, focusing on in-store experiences and their relationship with emotions, gender, and environmental awareness. Data were collected from 340 respondents through an online survey conducted between November 2024 and January 2025. The results indicate that feelings such as authenticity, fun, and interest in finding fashionable items are significantly associated with gender. Across all five dimensions, women perceive second-hand clothing shopping more positively than men. The biggest difference between the sexes is that women find this shopping more fun, enjoyable, and authentic. Men tend to be slightly more skeptical in their evaluation, with the lowest average score (2.65) on the question of whether shopping is “fun”. The study also reveals a strong correlation between the shopping experience and consumer attitudes. Individuals with prior experience in buying second-hand clothing exhibit greater environmental awareness, a stronger emotional connection with clothing, and a higher likelihood of participating in clothing swap events. Conversely, those without experience often harbor prejudices related to hygiene and perceive second-hand shopping as time-consuming and inconvenient. These findings highlight the importance of in-store experiences and the role of emotions in shaping consumer behavior towards second-hand clothing. The results have implications for retailers and policymakers seeking to promote sustainable consumption practices and enhance the appeal of the second-hand clothing market.

## 1. Introduction

People’s shopping habits vary depending on their age and level of education, but the fashion industry currently forms a significant part of their lives. In recent years, the consumer society has been constantly taking on new dimensions. Consumers buy surplus products because purchasing behavior depends primarily on emotions. In general, economics assumes that consumers make rational decisions (Bhui et al., 2021). However, the reality is far from this assumption. Consumers are rarely rational. Buying green (ecological) second-hand clothing can be considered rational purchasing behavior.

The production and consumption of clothing pose a significant burden on waste management and the environment (Lang & Zhang, 2019). To mitigate these negative impacts, sustainable consumption, which emphasizes the efficient use of resources and waste minimization, is increasingly being promoted (Borusiak et al., 2020; H. Park & Martinez, 2020). One recommended approach is to buy second-hand goods, which brings both environmental and financial benefits, such as reduced water consumption and production costs (Hur, 2020). According to WRAP, extending the life of clothing by nine months can reduce its environmental impact by approximately 20–30% (King & Wheeler, 2016).

Key factors influencing environmentally responsible shopping include, in particular, individual consumer characteristics, such as psychological factors (attitudes, values, emotions), habits and lifestyle (previous shopping behavior), and socio-demographic characteristics (age, gender, education) (C. S. Tan et al., 2017; Liang et al., 2019; Zhang & Dong, 2020) When purchasing clothing, consumers prioritize style and individuality, while when purchasing food, health plays a significant role (H. J. Park & Lin, 2020). There are also differences between online and brick-and-mortar shopping. Online shopping focuses on convenience, while brick-and-mortar stores emphasize quality service and qualified personnel.

The purpose is to explore Generation Z’s shopping behavior towards second-hand clothing in Slovakia, focusing on how in-store experiences, emotions, gender, and environmental awareness influence consumer attitudes.

This paper describes the behavior of Generation Z from Slovakia when buying worn clothes, focusing on gender, and the experience of buying second-hand clothes supporting environmental awareness and nostalgia using the analysis of categorical variables such as the Chi-square test and the correspondence analysis.

The paper aims to identify shopping behaviors in brick-and-mortar stores with second-hand clothing from the point of view of Generation Z. We formulate several scientific questions to achieve the aim:

Does gender affect the emotions of buying second-hand clothes in brick-and-mortar stores in Generation Z consumers?
Does gender affect the primary reasons for shopping, benefits, and obstacles when buying second-hand clothes in brick-and-mortar stores in Generation Z consumers?
Does gender affect the environmental awareness and emotional relationship to clothes in Generation Z consumers?
Does the experience of buying second-hand clothes affect Generation Z consumers’ emotions when buying second-hand clothes in brick-and-mortar stores?
Does the experience of buying second-hand clothes in brick-and-mortar stores affect the environmental awareness and emotional relationship to clothes in Generation Z consumers?
Does the experience of buying second-hand clothes in brick-and-mortar stores affect the shopping experience of swapping clothes in Generation Z consumers?

## 2. Literature Review

Research on online shopping often uses a cognitive approach (Kawaf & Tagg, 2017). Simanjuntak et al. (2020) propose the concept of “flow” to capture the customer experience in an online environment. In this state, customers lose track of time because they are fully focused on online navigation on a company’s website (Nica et al., 2022; Trevinal & Stenger, 2014). This work aims to analyze the factors that influence the purchase of second-hand clothing, with an emphasis on psychological and socio-demographic aspects.

Generations and their buying behavior. Consumers are most often divided by age into baby boomers, Generation X, Generation Y, and Generation Z in multigenerational marketing. This classification is based on date of birth, but many authors use different age ranges, with each group exhibiting similar buying characteristics (De Keyser et al., 2015). Technological developments have changed the buying behavior of consumers, especially Generation Z. This generation is characterized by digital knowledge (Malelak & Anastasia, 2020; Zulauf & Wagner, 2022), social awareness, and a desire for authenticity. As a result, Generation Z’s buying behavior differs from that of previous generations. One notable trend is the growing interest in second-hand brick-and-mortar stores. These consumers primarily consider sustainability, fashion style, price, and hygiene before purchasing second-hand clothing. Research has shown that students who buy second-hand clothing are more aware of environmental issues (Rahadhini et al., 2020). Research on Chinese consumers shows that young consumers are more concerned about environmental issues than older consumers (Xu, 2020). Consumers consider quality as one of the prerequisites for purchasing, with 6 out of 10 respondents prioritizing the quality of clothing and accessories over the price, and purchasing sustainable products is preferred. On the other hand, other studies on attitudes towards green consumption refute the hypothesis that young people have a greater social conscience (Sachdeva & Goel, 2015; Estévez et al., 2020).

Emotions play a significant role in shaping consumer behavior, influencing not only impulsive purchases but also long-term decision-making processes (Kuuru et al., 2020). Research indicates a complex interplay between various emotional states and shopping habits (Dragolea et al., 2023; Tarka et al., 2022). Specifically, negative emotions such as stress, disappointment, guilt, and envy can significantly impact consumer choices, often leading to undesirable and enduring consequences (Bejtkovský, 2016; Thangavel et al., 2021). Recognizing the power of emotions, marketers increasingly leverage experiential marketing strategies to forge deeper connections with consumers. By appealing to emotional experiences, brands aim to cultivate stronger bonds and encourage the sharing of these experiences (Yan et al., 2015; Munsch, 2021). This approach aligns with consumer expectations, as individuals demonstrably seek positive emotional experiences during their shopping journeys (PwC, 2018; Liang et al., 2019). Consequently, marketers strive to evoke positive emotions like joy, excitement, and happiness to enhance consumer satisfaction and brand loyalty.

Consumer demographics significantly influence the emotional drivers behind shopping behavior. Young people, for example, are known for their propensity towards impulsive purchases (Ortega-Egea et al., 2014). Middle-generation women, on the other hand, prioritize quality products at competitive prices, while simultaneously seeking emotional fulfillment from their shopping experiences (Kacprzak & Dziewanowska, 2019). In contrast, middle-aged and older men, particularly those with higher incomes, tend to make more unemotional purchases, focusing primarily on image and quality (Iravanian & Ravari, 2020).

The emotional landscape of shopping becomes particularly nuanced in the context of vintage and second-hand clothing. Rodrigues et al. (2023) identified nostalgia and fashion involvement as potent drivers for consumers who prefer second-hand apparel. Nostalgia, in this context, fuels a preference for items that evoke memories of past trends and a desire to recapture the feelings of earlier, perhaps more idealized, times (Evans et al., 2022). This highlights how emotional connections to the past can significantly influence consumption choices in the second-hand market.

In summary, the literature underscores the importance of emotions in understanding shopping behavior. Marketers strategically appeal to emotions to build brand loyalty, and consumers themselves seek positive emotional experiences. Demographic factors and specific product categories, such as second-hand clothing, further complicate the relationship between emotions and purchasing decisions.

The daily production of new products increases landfilling, which negatively affects the environment (Boström, 2020). Clothing gradually loses its value; consumers often do not even wear newly purchased clothes (Lichy et al., 2023). Second-hand goods play an important role, especially in the clothing industry. Companies collect, sort, and distribute clothing. Alternative shopping has the potential for a climate crisis (D’Adamo et al., 2022; Persson & Hinton, 2023). Buying used goods reduces the waste that harms the environment, and this way of shopping can be considered an alternative to buying expensive brands. Every purchase of used clothing contributes to the recycling system (Gray et al., 2022; Jain et al., 2021). One garment can be owned by several people during its lifetime. In addition to second-hand shopping, clothes can be exchanged at various events. Matthews and Hodges examined the benefits that participants gained from engaging in such events. Informants perceived that donating as part of the exchange allowed them to clean out their closets, recycle clothes, and gain instant gratification. At receptions, they stated that getting free stuff is a plus; they trusted the origin of the goods and welcomed the advice that they received (Camacho-Otero et al., 2020).

Generation Z represents a specific segment of consumers with distinct purchasing characteristics. The impact of technology, the growing interest in sustainability and product quality, as well as the conflicting results of research on the environmental awareness of young people are key aspects to consider when analyzing the purchasing behavior of this generation. No research in Slovakia has addressed the influence of emotions when shopping in second-hand stores by Generation Z.

## 3. Methodology

Sample. Table 1 divides the final sample of 340 respondents from Generation Z according to gender, residence size, and experience in buying second-hand clothes in brick-and-mortar stores. The input data are obtained from November 2023 to January 2024 via Google Forms, for 30 days. The online questionnaire was anonymously divided into several parts (see Appendix A). The questionnaire was developed based on a literature review on second-hand shopping behavior and sociodemographic influences. In our study, we applied a strict sample selection criterion by excluding all the respondents who did not belong to Generation Z. This decision was based on our research focus on the specific shopping behaviors of this generational group in the second-hand clothing market. The sample size was determined using a minimum sample size calculation based on a standard statistical power analysis to ensure methodological rigor. The exclusion of other generational groups was intentional to maintain the study’s internal validity and to avoid confounding effects related to generational differences in shopping behavior.

Table 2 describes the sample in detail in terms of several variables, not only from the point of view of gender, the size of residence, and shopping experience with second-hand clothes in brick-and-mortar stores. As we can see, 70 men and 97 women have experience in buying second-hand clothes with a potential interest in buying them in the future, as opposed to 82 respondents. These respondents have a negative attitude towards shopping in second-hand stores. Finally, we find that 84 respondents living in a village with less than 5000 inhabitants with experience in shopping in second-hand stores with a potential interest in another purchase in the future represent the largest subgroup in the sample, together with 49 respondents living in a city with more than 20,000 inhabitants.

Variables. Table 2 shows the categorical (nominal, ordinal) variables dealing with sociodemographic characteristics: the shopping experience with second-hand clothes in brick-and-mortar stores; preferred brick-and-mortar stores; the advantages and disadvantages of buying second-hand clothes; and selected characteristics describing second-hand clothing shopping in brick-and-mortar stores as authentic, liberating, pleasuring, fun, and interesting shopping. Table 2 reveals that more than 56% of the final sample is male, almost 71% of the respondents have a secondary education, and almost 50% of the respondents live in a village with less than 5000 inhabitants. In addition, we find that 167 consumers (almost 50%) have experienced shopping for second-hand clothing and plan to continue shopping in brick-and-mortar stores. On the other hand, almost 25% of respondents have a negative attitude towards buying second-hand clothes in brick-and-mortar stores. Humana People to People (almost 33%) and Textile House (more than 10%) are the most preferred brick-and-mortar clothing stores in the second-hand segment. Finally, we find that shopping for second-hand clothes in brick-and-mortar stores brings fun and pleasure to the majority of respondents (more than 65% of all). Moreover, shopping in brick-and-mortar second-hand stores is definitely or rather authentic (more than 72% of respondents), liberating (more than 70%), and interesting (more than 66%). The key benefit of buying second-hand clothes is the price for most respondents, but almost 20% of consumers are aware of the environmental impact of clothing production on the environment. On the other hand, the main obstacles when shopping in second-hand stores are primarily worn clothing (almost 42%) and the time-consuming search for clothes (more than 20%).

Hypotheses. Based on research questions, we formulate hypotheses.

Does gender affect the emotions of buying second-hand clothes in brick-and-mortar stores in Generation Z consumers?

▪ Gender and the authenticity level of buying SHC are independent.
▪ Gender and the freedom level of buying SHC are independent.
▪ Gender and the pleasure level of buying SHC are independent.
▪ Gender and the fun level of buying SHC are independent.
▪ Gender and the interest level in buying SHC are independent.

Does gender affect the primary reasons for shopping, benefits, and obstacles when buying second-hand clothes in brick-and-mortar stores in Generation Z consumers?

▪ Gender and the primary reasons for buying SHC are independent.
▪ Gender and the benefits of buying SHC are independent.
▪ Gender and the disadvantages of buying SHC are independent.

Does gender affect the environmental awareness and emotional relationship to clothes in Generation Z consumers?

▪ Gender and building an emotional relationship with clothes are independent.
▪ Gender and the consideration of the future consequences of buying clothes and shoes are independent.
▪ Gender and the awareness of the environmental impact of buying clothes and shoes are independent.

Does the experience of buying second-hand clothes affect Generation Z consumers’ emotions when buying second-hand clothes in brick-and-mortar stores?

▪ Experience with shopping for SHC in brick-and-mortar stores and the degree of authenticity are independent.
▪ Experience with shopping for SHC in brick-and-mortar stores and the degree of freedom are independent.
▪ Experience with shopping for SHC in brick-and-mortar stores and the degree of pleasure are independent.
▪ Experience with shopping for SHC in brick-and-mortar stores and the level of fun are independent.
▪ Experience with shopping for SHC in brick-and-mortar stores and the level of interest are independent.

Does the experience of buying second-hand clothes in brick-and-mortar stores affect the environmental awareness and emotional relationship to clothes in Generation Z consumers?

▪ Experience with shopping for SHC in brick-and-mortar stores and environmental awareness are independent.
▪ Experience with shopping for SHC in brick-and-mortar stores and the emotional relationship with the clothes are independent.

Does the experience of buying second-hand clothes in brick-and-mortar stores affect the shopping experience of swapping clothes in Generation Z consumers?

▪ Experience with shopping for SHC in brick-and-mortar stores and the shopping experience of swapping clothes are independent.

Methods. This research primarily explores the emotions associated with shopping for second-hand clothing from the perspective of Generation Z. It examines the relationship between feelings of authenticity, freedom, pleasure, fun, and interest with factors such as gender, previous experience with second-hand shopping, and other relevant aspects.

The Chi-square test and the correspondence analysis are statistical methods used to analyze the relationship between categorical variables. The Chi-square test evaluates whether a statistically significant association exists by comparing the observed and expected frequencies in a contingency table. Meanwhile, the correspondence analysis graphically represents relationships between two or more categorical variables in a two-dimensional space.

The Chi-square test consists of multiple steps, including creating a contingency table with observed frequencies, computing the expected frequencies, determining the *p*-value, and analyzing the findings.

(1) $$\chi^{2}=\sum \frac{{\left(O_{i}-E_{i}\right)}^{2}}{E_{i}}$$

where:

$\chi^{2}$ Chi-square derived from a pivot table,
$O_{i}$ observed (actual) frequencies,
$E_{i}$ expected frequencies.

The test requires categorical variables (nominal or ordinal), a random sample, independence between the two categorical variables, and a sufficiently large sample. Additionally, for a 2 × 2 contingency table, all the expected counts must be at least 5. In larger tables, at least 80% of the expected frequencies should be 5 or higher to maintain the test’s validity.

Cramer’s *V* quantifies the strength of the association between two categorical variables. This statistical metric ranges from 0 to 1, where 0 indicates no relationship and 1 denotes a perfect association.

(2) $$V=\sqrt{\frac{\chi^{2}}{n\times \min\left(k-1,\;r-1\right)}}$$

where:

$\chi^{2}$ Chi-square derived from a pivot table,
*n* total number of observations,
*k* number of columns in the pivot table,
*r* number of rows in the pivot table.

Cramer’s *V* is categorized into four levels:

▪ below 0.1—weak dependence,
▪ 0.1–0.3—moderately strong addiction,
▪ 0.3–0.5—strong dependence,
▪ above 0.5—very strong association.

When a statistically significant relationship between categorical variables is found, the correspondence analysis is used to visualize these relationships. This statistical method examines the dependence between two categorical variables by analyzing their categories within a contingency table. The process consists of four essential steps: creating a contingency table with absolute frequencies, creating a correspondence table with relative or expected frequencies, reducing the dimensions, and visualizing the results using a correspondence map.

The absolute frequencies *n_ij_* serve as the input matrix for determining the row absolute and column marginal frequencies in the correspondence matrix. The relative frequency is calculated with the row profiles *p_i_*_/*j*_ and the column profiles *p_j_*_/*i*_ (Štefancová et al., 2022).

(3) $$p_{j/i}=\frac{n_{ij}}{n_{i+}}=\frac{p_{ij}}{p_{+j}}$$

(4) $$p_{ij}=\frac{n_{ij}}{n_{+j}}=\frac{p_{ij}}{p_{+j}}$$

The relative positioning of the points in the dimensional space indicates the strength of dependence. When the points are closely clustered in the correspondence matrix, it indicates a strong relationship. The total inertia (I) represents the variability of the points and is calculated using the following Formula (5) (Ali; Doey; Hanada) (Ali et al., 2018) (McHugh, 2013).

(5) $$I^{2}=\sum_{i}p_{+j}d_{j}^{2}$$

The correspondence analysis is performed using IBM SPSS 29 (Mustafy & Rahman, 2024) with data gathered from an online questionnaire. The output is a map that visually represents the relationship between the categories examined. However, the correspondence analysis is relevant provided that the contingency is statistically significant based on the Chi-square test. Unlike many statistical methods, the correspondence analysis does not require the assumption of a normal distribution. However, it does have specific assumptions, such as the homogeneity of variance for the row and column variables, the data must be discrete, the dataset should contain at least three categories of categorical variables, and all the frequencies in the contingency table must be non-negative (Ali et al., 2018).

## 4. Results

Generation Z and their emotions in shopping for clothes in the second-hand segment. Table 3 demonstrates that gender and the characteristics of shopping for second-hand clothes, like authenticity, fun, and interest, are statistically significantly dependent (*p*-value less than 0.05), unlike freedom and pleasure.

First, Table 4 demonstrates that most women (almost 77%) consider buying second-hand clothes to be authentic; similarly, more than 69% of men share the same opinion. Second, less than 39% of women think that shopping for second-hand clothes is definitely liberating. On the other hand, most men find shopping for second-hand clothes rather liberating. Third, most men (more than 36%) and women (less than 42%) think that shopping for second-hand clothes brings definite pleasure. Fourth, both men and women definitely think that second-hand shopping is fun and interesting.

**Figure 1:**
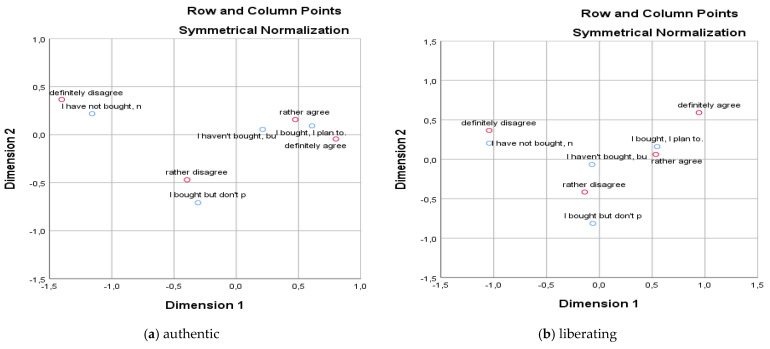

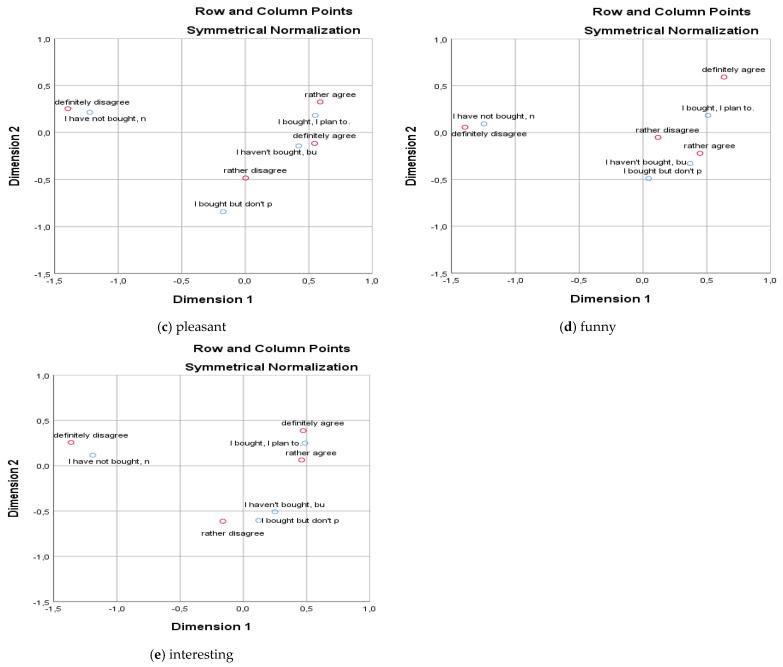
Correspondence maps I.

Table 5 and Figure 1 show that the consumer’s experience with shopping for second-hand clothes in brick-and-mortar stores affects the measure of the characteristics associated with shopping in second-hand stores. Shopping for second-hand clothes can bring authenticity, freedom, pleasure, fun, and interest. Generation Z brings different opinions and attitudes based on their experience with buying second-hand clothes. We find that the degree of authenticity, freedom, pleasure, fun, and interest, and the shopping experience with second-hand clothing is dependent. We reject the null hypothesis (*p*-value is less than 0.05). All these dependencies are moderately strong except for two dependencies based on Cramer’s *V*.

Generation Z and their shopping behavior in the second-hand segment. Table 6 shows that there is no dependence between gender and the primary reason for shopping, and the advantages and disadvantages of buying second-hand clothes or shoes in contrast to the preferred online second-hand platforms. We do not reject the null hypothesis (*p*-value is higher than 0.05). On the other hand, gender affects building an emotional relationship with clothes, considering the potential consequences resulting from buying clothes, but also environmental awareness (*p*-values less than 0.05). We reject the null hypothesis and accept the alternative hypothesis. Cramer’s *V* demonstrates that there is a weak dependence between the variables.

**Figure 2:**
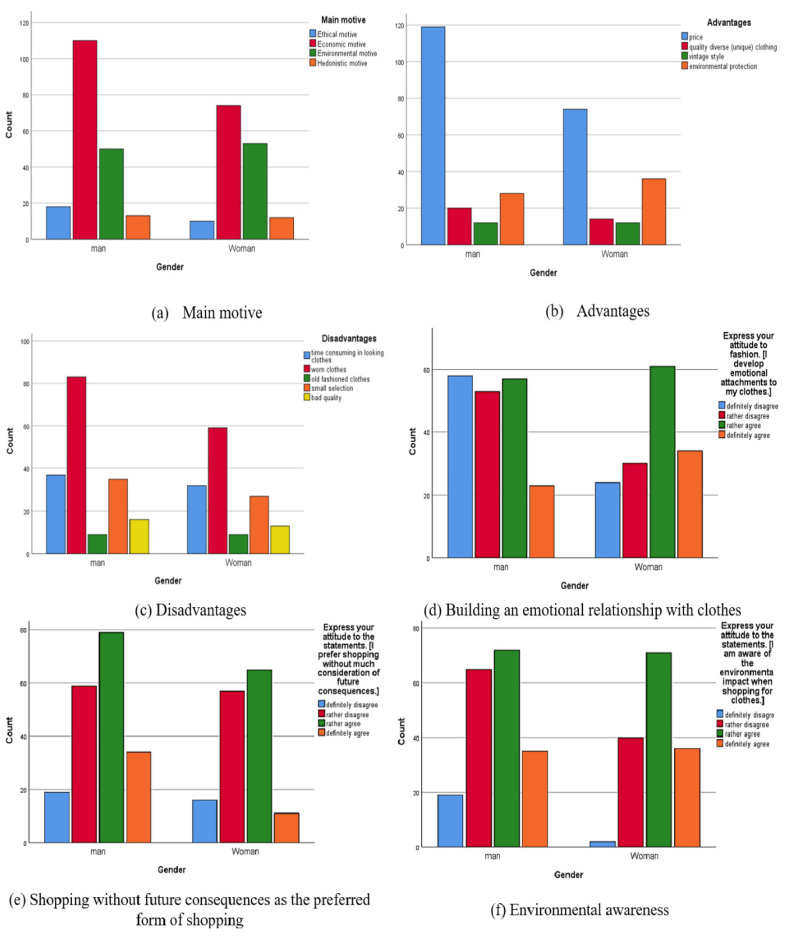
Bar chart.

Figure 2a shows that second-hand shopping primarily provides economic benefits and environmental protection against the overproduction of new clothing for traditional stores with first-hand clothes. Figure 2b reveals that price and environmental protection are the main benefits of buying second-hand clothes in contrast to high-quality diverse (unique) clothes and vintage clothes, regardless of gender. On the other hand, Figure 2c demonstrates that worn clothes, the time-consuming search for clothes, and fewer choices with other disadvantages are ranked equally regardless of gender. Moreover, we find that women tend to build an emotional relationship with clothes more than men. On the other hand, men prefer shopping without future consequences more than women. However, women are more environmentally conscious than men.

Table 7 shows that there is a statistically significant relationship between the online shopping experience of second-hand clothes and environmental awareness, respectively building an emotional relationship with clothes and swapping clothes. We reject the null hypothesis and accept the alternative hypothesis (*p*-value less than 0.05). The results show that there is a weak dependence between the variables based on Cramer’s *V*.

**Figure 3:**
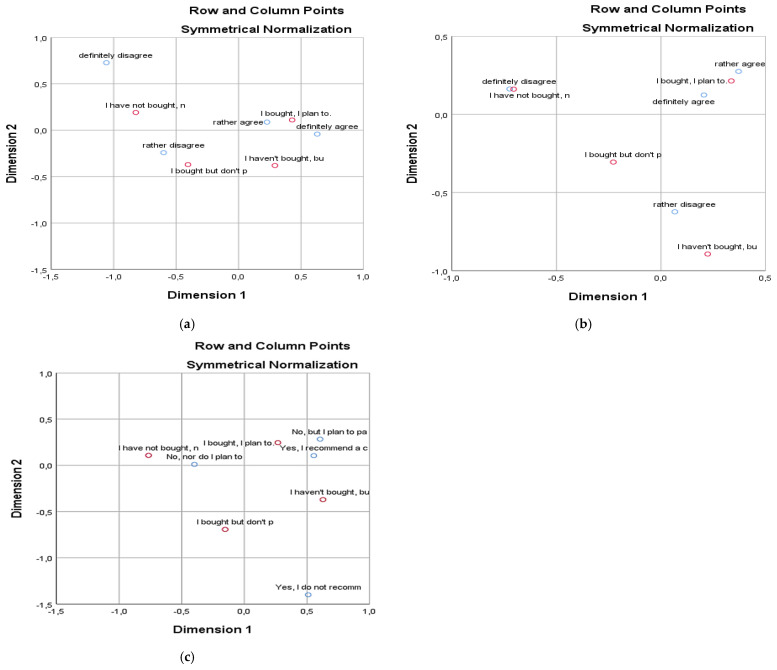
Correspondence maps II. (**a**) Experience with shopping for second-hand clothes in brick-and-mortar stores and environmental awareness are independent. (**b**) Experience with shopping for second-hand clothes in brick-and-mortar stores and the emotional relationship with the clothes are independent. (**c**) Experience with shopping for second-hand clothes in brick-and-mortar stores and the shopping experience of swapping clothes are independent.

Figure 3a shows that experienced consumers planning a future purchase of second-hand clothing are more aware of the environmental consequences of the continuous production of surplus clothing, similar to consumers considering the first purchase of second-hand clothing in a brick-and-mortar store. On the other hand, skeptical customers without shopping experience with second-hand clothes are more or less aware of the negative consequences of unsustainable consumption on the environment. The results show that Generation Z consumers buying second-hand clothes in the future are also environmentally aware, and aware of the negative consequences of unsustainable consumption on the environment. Other groups of consumers, depending on the shopping experience with second-hand clothes in brick-and-mortar stores, tend not to have an environmental sense, except for consumers planning their first future purchase of second-hand clothes in brick-and-mortar stores. 

Figure 3b shows that Generation Z consumers with experience in shopping for second-hand clothing and an interest in future purchases mostly form an emotional relationship with clothing, unlike other groups. The results show that consumers buying second-hand clothes with a potential interest in future purchases generally develop an emotional relationship with their clothes. On the other hand, two groups of consumers depending on the experience of shopping for second-hand clothes, such as consumers with potential interest without any experience of shopping for second-hand clothes and consumers with the experience of shopping for second-hand clothes without planning a future purchase, rather build their emotional relationship with the clothes. Finally, consumers with a negative preference for buying second-hand clothes are indifferent to their clothes: they have no emotional connection to the clothes and no nostalgia.

Figure 3c shows that most consumers have no experience in swapping clothes, regardless of their experience in buying second-hand clothes in brick-and-mortar stores. Moreover, we find that consumers with a cheerful outlook towards buying second-hand clothes in brick-and-mortar stores also have the greatest experience with swapping clothes, unlike other consumers. The results show that Generation Z consumers buying second-hand clothes with a potential interest in future purchases also have the experience of exchanging clothes at various swap events. Moreover, this group has a positive experience and recommends this form of sustainable consumption. On the other hand, consumers who are skeptical about buying second-hand clothes in brick-and-mortar stores also have a negative attitude towards participating in events promoting clothing exchange.

## 5. Discussion

The study finds that shopping for second-hand clothing brings authenticity and pleasure, but also interest in finding fashionable clothing among young consumers. Gender affects the building of an emotional relationship with clothing and environmental awareness. Women are more environmentally friendly than men. On the other hand, gender does not affect the primary reason for shopping for second-hand clothing, and the benefits and barriers in the brick-and-mortar stores. Another group of consumers, according to the shopping experience of buying second-hand clothes in brick-and-mortar stores, are not environmentally conscious, unlike consumers planning their first purchase of second-hand clothes in brick-and-mortar stores.

From the point of view of the shopping experience, Generation Z considers price and environmental protection to be the main motivators. The results show that consumers with experience in buying second-hand clothes with a potential interest in future purchases also have experience in exchanging clothes at swap events. On the other hand, consumers without experience in buying second-hand clothes without planning a future purchase have prejudices against used clothes due to hygiene, the time-consuming search, and the smaller selection. These consumers also have a negative attitude towards swapping clothes at swap events; moreover, their attitude towards clothes is indifferent without building any emotional attachment.

All of these findings provide valuable implications for second-hand clothing managers or retailers.

Emotions from shopping. Shopping for second-hand clothing is associated with positive emotions such as authenticity, liberation, and pleasure. Women generally exhibit stronger emotional connections with clothing and are more environmentally conscious than men. The majority of women (almost 77%) consider buying used clothes to be authentic; similarly, 69% of men share the same opinion. Shopping for used clothes is fun for almost 61% of men and 73% of women. Gender affects the development of an emotional relationship with clothing, considering the potential consequences of purchasing clothing and environmental awareness. Utilitarian values, emotional values, and social values are likely to influence consumer attitudes toward sustainable fashion (Chi, 2015; H. J. Park & Lin, 2020). Price and environmental protection are the primary motivators for purchasing second-hand clothes. Generation Z, particularly those with prior experience, are more environmentally conscious and driven by sustainability considerations. In their research, Reis et al. showed the influence of nostalgia when shopping, in which they were exposed to two states of nostalgia (nostalgia, present) and two own states (I, others). The results showed that young adults did not prefer second-hand clothes when nostalgic feelings prevailed, but a significant effect on attitudes and perceptions was observed (Reis, 2020). Positive emotions have a significant effect on impulse buying regardless of gender (Pramestya & Widagda, 2020). Other previous studies show that women are more involved in buying second-hand products than men (Li et al., 2022; Turunen et al., 2020). There is an assumption of a stronger emotional feeling towards the surrounding environment. Van der Horst (2024) demonstrates that women are more sensitive to environmental problems than men. Women are more prosocial, altruistic, and empathetic than men (Witek & Kuźniar, 2020). The results of a Chinese study show that the public in China have a positive attitude towards green consumption, and women from developed regions are more interested in ecological consumption. In the study (Huang et al., 2022), they identified five psychological consequences related to emotions when buying second-hand products, such as uniqueness and the strengthening of creativity and individuality, the feeling of satisfaction from a bargain purchase, the feeling of joy from the surroundings, the feeling of help provided, and feeling good about oneself.

The reasons for shopping for second-hand clothes. Generation Z has different attitudes towards buying second-hand clothes based on their experience. Those who plan to buy second-hand clothes in the future are environmentally conscious and aware of the consequences of unsustainable consumption on the environment (Hur, 2020). Our results show that second-hand shopping primarily brings economic benefits and environmental protection. Moreover, 30% more men than women consider economic reasons as the main motive. In conclusion, women prefer environmental benefits more than men. However, the price was the main benefit for both sexes. On the other hand, worn clothes are the biggest disadvantage. Our research also shows that changing clothes is the closest thing to people with a cheerful outlook on shopping, especially in brick-and-mortar stores. Through a holistic interpretation of the results, the authors of the study (Karpova et al., 2022) discovered two critical factors determining the overall satisfaction and success of the temporary exchange: (1) the closeness of the relationship between the exchange partners; and (2) the participants’ love of clothing.

Nowadays, young consumers think that these stores are beneficial and cool, although the original motives were different (Khandual & Pradhan, 2019). The literature provides explanations of the reasons for second-hand consumption. Hygiene concerns, time-consuming searches, and the limited selection are perceived as major barriers to entry for those who are new to second-hand shopping. Second-hand consumption motives are primarily driven by individual satisfaction as part of the motivation theory (Long & Suomi, 2022). Akgün and Mezde (2024) divide the reasons for buying second-hand goods into three categories: critical, recreational, and economic. Sustainability and economic motives have a positive effect on the attitude towards second-hand shopping and the distance from the consumer system (T. M. Tan et al., 2022).

The reasons for purchasing goods may be related to the experience of shopping online or in a brick-and-mortar store. In addition, prior online second-hand shopping experience has a positive moderate effect on the relationship between perceived sustainability and the distance from the consumption system (Ek Styvén & Mariani, 2020). Consumers are sometimes prevented from buying second-hand products by the mistrust of online stores, due to the low reliability and quality of the products (Calvo-Porral et al., 2024). However, online stores also bring disadvantages, such as shipping and handling fees, exchange or refund for goods, after-purchase services, and uncertainty about the delivery of the ordered goods (Sendow et al., 2019). One of the main benefits of brick-and-mortar shopping is the shopping experience itself, as the brick-and-mortar consumer can touch the product. This sensory experience provides confidence in deciding on the selected product (Mateos-Mínguez et al., 2021; Moes & van Vliet, 2017). The in-store experience plays a crucial role, with factors like the store atmosphere and the ability to physically examine items influencing purchasing decisions. Additionally, brick-and-mortar stores provide professional salespeople (Good & Hyman, 2020; Rapp et al., 2015). Personal opinions on second-hand stores may vary. According to Sorensen (Sorensen & Jorgensen, 2019), customers find stores unpleasant because of the unpleasant smell and the disorganization in the store, so some customers prefer to visit an online platform offering second-hand clothes.

Theoretical implications. This research provides valuable insights into the emotional and behavioral dynamics of Generation Z’s SHC shopping habits. It highlights the importance of emotional connections and environmental awareness in driving sustainable consumption practices. The study contributes to a deeper understanding of the factors influencing Generation Z’s engagement with the circular economy. The study shows the relationships between the consumer experience, an emotional connection to clothing, and environmental consciousness.

Practical Implications. Second-hand retailers can leverage the emotional aspects of shopping (authenticity, fun, interest) to attract Generation Z consumers. Highlighting the environmental benefits of second-hand shopping can appeal to environmentally conscious consumers. Addressing concerns about worn clothing and time-consuming searches (e.g., through curated selections or improved store layouts) can enhance the shopping experience. The study underscores the potential of second-hand shopping as a sustainable consumption practice. Promoting clothing swaps and raising awareness about the environmental impact of fast fashion can further encourage sustainable behaviors. This study provides a framework for future research on consumer behavior in the second-hand clothing market, particularly among Generation Z. The methodology used can be applied to investigate other aspects of sustainable consumption.

Limitations. Limited sample size and scope: The study’s findings are based on a sample of 340 young consumers in Slovakia, which may not be representative of the broader population. The focus on young consumers limits the generalizability of the findings to other age groups. Focus on a specific consumer segment: The study primarily focuses on existing and potential second-hand shoppers, neglecting the perspective of non-consumers. Lack of a deeper exploration of certain factors: While the study touches upon factors like nostalgia and impulse buying, a more in-depth exploration of these influences would be beneficial.

Future research should include a broader range of consumer segments, including older generations, different socioeconomic groups, and non-consumers, to gain a more comprehensive understanding of the market. Conducting comparative studies across different countries and cultures would provide valuable insights into the global trends and variations in second-hand clothing consumption. Longitudinal studies would help to track changes in consumer attitudes and behaviors over time, particularly in response to evolving trends in sustainability and fashion. Moreover, the role of online platforms and social media in shaping second-hand clothing consumption needs further investigation.

Research should delve deeper into the interplay between the social and environmental factors that influence consumer choices, such as social norms, peer pressure, and environmental awareness campaigns.

## 6. Conclusions

This study provides valuable insights into the motivations, attitudes, and behaviors of young consumers towards second-hand clothing. We find that shopping in brick-and-mortar second-hand stores brings fun and pleasure to experienced second-hand clothing shoppers with a potential interest in future purchases. Moreover, these consumers say that shopping for second-hand clothes is authentic, liberating, and interesting, unlike consumers without any experience or interest in a future purchase. The results show that gender impacts purchasing behavior, as authenticity, fun, and interest together with gender are statistically significant dependent based on the Chi-square test, unlike other characteristics such as freedom and pleasure. Finally, we find that the experience of shopping for clothes in brick-and-mortar stores depends on environmental awareness and building an emotional relationship with clothes. Regarding planning and purchasing in the future, Generation Z is more aware of the negative impacts of the fashion and textile industry on the environment. These consumers feel nostalgic about their clothes, unlike other groups depending on the experience of shopping in the second-hand segment. Finally, the study also demonstrates that experienced consumers planning to purchase used clothing in the future participate in swap events. This group of consumers does not avoid alternative shopping methods to traditional first-hand clothing stores. However, further research is necessary to address the identified limitations and gain a more comprehensive understanding of this evolving market. By addressing these research gaps, we can better understand the drivers of sustainable consumption and develop effective strategies to promote the growth of the second-hand clothing market.

## Figures and Tables

**Table 1:** Sample.

Gender	Residence Size	Experience in Shopping for SHC in Any Brick-and-Mortar Store				Total
		I Have Not Bought, nor Do I Plan To	I Have Not Bought It, but I Plan To	I Bought It, I Plan To	I Bought It but Do Not Plan to Anymore	
Man	up to 5000	30	13	33	10	86
	5001–20,000	10	3	12	6	31
	more than 20,000	23	13	25	13	74
Total		63	29	70	29	191
Woman	up to 5000	9	5	51	9	74
	5001–20,000	4	3	22	2	31
	more than 20,000	6	4	24	10	44
Total		19	12	97	21	149
Total	up to 5000	39	18	84	19	160
	5001–20,000	14	6	34	8	62
	more than 20,000	29	17	49	23	118
Total		82	41	167	50	340

**Table 2:** Variables.

Variable	Acronym	Category	N	%	Modus	Median
Gender	0	Man	191	56.2	0	0
	1	Woman	149	43.8		
Education level	0	Elementary education	25	7.4	1	1
	1	Secondary education	240	70.6		
	2	University education	75	22.1		
Residence size	0	Up to 5000 inhabitants	160	47.1	0	1
	1	5001–20,000 inhabitants	62	18.2		
	2	More than 20,000 inhabitants	118	34.7		
Experience with shopping for SHC in any brick-and-mortar store	0	I have not bought, nor do I plan to.	82	24.1	2	2
	1	I have not bought it, but I plan to.	41	12.1		
	2	I bought it, I plan to.	167	49.1		
	3	I bought it, but do not plan to anymore.	50	14.7		
Preferred second-hand brick-and-mortar store	0	Textile House	38	11.2	3	2
	1	Humana People to People Slovakia	111	32.6		
	2	Local second-hand shop	32	9.4		
	3	None	159	46.8		
The main advantage of shopping for SHC	0	Price	193	56.8	0	0
	1	Quality diverse (unique) clothing	34	10.0		
	2	Vintage style	24	7.1		
	3	Environmental protection	64	18.8		
	4	Other	25	7.4		
The main disadvantage of shopping for SHC	0	Time-consuming to look for clothes	69	20.3	1	1
	1	Worn clothes	142	41.8		
	2	Old fashioned clothes	18	5.3		
	3	Small selection	62	18.2		
	4	Bad quality	29	8.5		
	5	Other	20	5.9		
Shopping for SHC is authentic.	0	I disagree.	52	15.29	3	2
	1	I rather disagree.	45	13.24		
	2	I rather agree.	89	26.18		
	3	I agree.	157	46.18		
Shopping for SHC is liberating.	0	I disagree.	71	20.88	2	2
	1	I rather disagree.	29	8.53		
	2	I rather agree.	121	35.59		
	3	I agree.	119	35.00		
Shopping for SHC is pleasing	0	I disagree.	66	19.41	3	2
	1	I rather disagree.	27	7.94		
	2	I rather agree.	116	34.12		
	3	I agree.	131	38.53		
Shopping for SHC is fun.	0	I disagree.	68	20.00	3	2
	1	I rather disagree.	47	13.82		
	2	I rather agree.	108	31.76		
	3	I agree.	117	34.41		
Shopping for SHC is interesting.	0	I disagree.	61	17.94	3	2
	1	I rather disagree.	52	15.29		
	2	I rather agree.	74	21.76		
	3	I agree.	153	45.00		

Note: second-hand clothes (SHC).

**Table 3:** Hypotheses I.

Null Hypothesis	Pearson Chi-Square	Df	*p*-Value	N
Gender and authenticity level of buying SHC are independent.	10.655 a	3	0.014	340
Gender and freedom level of buying SHC are independent.	4.835 b	3	0.184	340
Gender and pleasure level of buying SHC are independent.	4.887 c	3	0.180	340
Gender and fun level of buying SHC are independent.	14.362 d	3	0.002	340
Gender and interest level in buying SHC are independent.	14.475 e	3	0.002	340

Note: second-hand clothes (SHC). (a) 0 cells (0.0%) have an expected count of less than 5. The minimum expected count is 18.41; (b) 0 cells (0.0%) have an expected count of less than 5. The minimum expected count is 12.71; (c) 0 cells (0.0%) have an expected count of less than 5. The minimum expected count is 11.83; (d) 0 cells (0.0%) have an expected count of less than 5. The minimum expected count is 20.60; (e) 0 cells (0.0%) have an expected count of less than 5. The minimum expected count is 22.79.

**Table 4:** What is shopping for second-hand clothes like according to gender? (N, %).

Shopping for Second-Hand Clothes Is	Gender	Disagree		Rather Disagree		Rather Agree		Agree		Sum	Mean	Agree		Disagree	
		N	%	N	%	N	%	N	%			N	%	N	%
Authentic	M	39	20.42	20	10.47	52	27.23	80	41.88	191	2.91	132	69.11	59	30.89
	F	13	8.72	22	14.77	37	24.83	77	51.68	149	3.19	114	76.51	35	23.49
Liberating	M	47	24.61	14	7.33	69	36.13	61	31.94	191	2.75	130	68.06	61	31.94
	F	24	16.11	15	10.07	52	34.90	58	38.93	149	2.97	110	73.83	39	26.17
Pleasing	M	45	23.56	14	7.33	63	32.98	69	36.13	191	2.82	132	69.11	69	30.89
	F	21	14.09	13	8.72	53	35.57	62	41.61	149	3.05	115	77.18	34	22.82
Funny	M	52	27.23	23	12.04	55	28.80	61	31.94	191	2.65	116	60.73	75	39.27
	F	16	10.74	24	16.11	53	35.57	56	37.58	149	3.00	109	73.15	40	26.85
Interesting	M	44	23.04	21	10.99	47	24.61	79	41.36	191	2.84	126	65.97	65	34.03
	F	17	11.41	31	20.81	27	18.12	74	49.66	149	3.06	101	67.79	48	32.21

**Table 5:** Hypotheses II.

Null Hypothesis	Pearson Chi-Square	Df	*p*-Value	Cramer’s *V*	N
Experience with shopping for SHC in brick-and-mortar stores and the degree of authenticity are independent.	97.418 a	9	0.000	0.309	340
Experience with shopping for SHC in brick-and-mortar stores and the degree of freedom are independent.	62.099 b	9	0.000	0.247	340
Experience with shopping for SHC in brick-and-mortar stores and the degree of pleasure are independent.	103.818 c	9	0.000	0.319	340
Experience with shopping for SHC in brick-and-mortar stores and the level of fun are independent.	92.840 d	9	0.000	0.302	340
Experience with shopping for SHC in brick-and-mortar stores and the level of interest are independent.	76.460 e	9	0.000	0.279	340

Note: second-hand clothes (SHC). (a) 0 cells (0.0%) have an expected count of less than 5. The minimum expected count is 5.06; (b) 2 cells (12.5%) have an expected count of less than 5. The minimum expected count is 3.50; (c) 2 cells (12.5%) have an expected count of less than 5. The minimum expected count is 3.26; (d) 0 cells (0.0%) have an expected count of less than 5. The minimum expected count is 5.67; (e) 0 cells (0.0%) have an expected count of less than 5. The minimum expected count is 6.27.

**Table 6:** Hypotheses III.

Null Hypothesis	Pearson Chi-Square	df	*p*-Value	Cramer’s *V*	N
Gender and the primary reasons for buying SHC are independent.	4.334 a	3	0.228	0.113	340
Gender and the benefits of buying SHC are independent.	6.808 b	3	0.078	0.147	315
Gender and the disadvantages of buying SHC are independent.	0.773 c	4	0.942	0.049	320
Gender and building an emotional relationship with clothes are independent.	17.813 d	3	0.000	0.229	340
Gender and the consideration of future consequences of buying clothes and shoes are independent.	8.347 e	3	0.039	0.157	340
Gender and the awareness of the environmental impact of buying clothes and shoes are independent.	14.773 f	3	0.002	0.208	340

Note: second-hand clothes (SHC). (a) 0 cells (0.0%) have an expected count of less than 5. The minimum expected count is 10.96; (b) 0 cells (0.0%) have an expected count of less than 5. The minimum expected count is 10.36; (c) 0 cells (0.0%) have an expected count of less than 5. The minimum expected count is 7.88; (d) 0 cells (0.0%) have an expected count of less than 5. The minimum expected count is 24.98; (e) 0 cells (0.0%) have an expected count of less than 5. The minimum expected count is 15.34; (f) 0 cells (0.0%) have an expected count of less than 5. The minimum expected count is 9.20.

**Table 7:** Hypotheses IV.

Null Hypothesis	Pearson Chi-Square	Df	*p*-Value	Cramer’s *V*	N
Experience with shopping for SHC in brick-and-mortar stores and environmental awareness are independent.	29.580 a	9	0.001	0.170	340
Experience with shopping for SHC in brick-and-mortar stores and the emotional relationship with the clothes are independent.	22.989 b	9	0.006	0.150	340
Experience with shopping for SHC in brick-and-mortar stores and the shopping experience of swapping clothes are independent.	22.435 c	9	0.008	0.148	340

Note: second-hand clothes (SHC). (a) 2 cells (12.5%) have an expected count of less than 5. The minimum expected count is 2.53; (b) 0 cells (0.0%) have an expected count of less than 5. The minimum expected count is 6.87; (c) 3 cells (18.8%) have an expected count of less than 5. The minimum expected count is 2.17.

## Data Availability

Data are contained within the article.

## Appendix A

Demographic Information

1. What is your gender?

☐ Male
☐ Female

2. What is your highest level of education?

☐ Elementary education
☐ Secondary education
☐ University degree

3. What is the size of your place of residence?

☐ Up to 5000 inhabitants
☐ 5001–20,000 inhabitants
☐ More than 20,000 inhabitants

Experience with second-hand clothing (SHC) shopping

4. Have you ever bought second-hand clothing or shoes in a brick-and-mortar store?

☐ I have not bought, nor do I plan to.
☐ I have not bought it, but I plan to.
☐ I bought it, I plan to.
☐ I bought it, but do not plan to anymore.

5. Which type of second-hand brick-and-mortar store do you prefer the most?

☐ Textile House
☐ Humana People to People Slovakia
☐ Local second-hand shop
☐ None

Perceptions of second-hand shopping

6. What do you consider the main advantage of shopping for second-hand clothing?

☐ Price
☐ Quality diverse (unique) clothing
☐ Vintage style
☐ Environmental protection
☐ Other

7. What do you consider the main disadvantage of shopping for second-hand clothing?

☐ Time-consuming to look for clothes
☐ Worn clothes
☐ Small selection
☐ Bad quality
☐ Other

Emotional perception of second-hand clothing shopping (indicate how much you agree with the following statements.)

8. Shopping for second-hand clothing is an authentic experience.

☐ I disagree.
☐ I rather disagree.
☐ I rather agree.
☐ I agree.

9. Shopping for second-hand clothing is liberating.

☐ disagree.
☐ I rather disagree.
☐ I rather agree.
☐ I agree.

10. Shopping for second-hand clothing is pleasing.

☐ I disagree.
☐ I rather disagree.
☐ I rather agree.
☐ I agree.

11. Shopping for second-hand clothing is fun.

☐ I disagree.
☐ I rather disagree.
☐ I rather agree.
☐ I agree.

12. Shopping for second-hand clothing is interesting.

☐ I disagree.
☐ I rather disagree.
☐ I rather agree.
☐ I agree.

## References

[B1-behavsci-15-00413] Akgün V. Ö., Mezde A. (2024). The impact of consumer motivation on repurchase in online second-hand shopping experience: An application on aimed at measuring university student tendencies. Third Sector Social Economic Review.

[B2-behavsci-15-00413] Ali F., Dissanayake D., Bell M., Farrow M. (2018). Investigating car users’ attitudes to climate change using multiple correspondence analysis. Journal of Transport Geography.

[B3-behavsci-15-00413] Bejtkovský J. (2016). The employees of the baby boomers generation, generation X, generation Y and generation Z in selected Czech corporations as conceivers of development and competitiveness in their corporation. Journal of Competitiveness.

[B4-behavsci-15-00413] Bhui R., Lai L., Gershman S. J. (2021). Resource-rational decision making. Current Opinion in Behavioral Sciences.

[B5-behavsci-15-00413] Borusiak B., Szymkowiak A., Horska E., Raszka N., Żelichowska E. (2020). Towards building sustainable consumption: A study of second-hand buying intentions. Sustainability.

[B6-behavsci-15-00413] Boström M. (2020). The social life of mass and excess consumption. Environmental Sociology.

[B7-behavsci-15-00413] Calvo-Porral C., Orosa-González J., Viejo-Fernández N. (2024). Barriers to online second-hand purchase behavior. Marketing Intelligence & Planning.

[B8-behavsci-15-00413] Camacho-Otero J., Pettersen I. N., Boks C. (2020). Consumer engagement in the circular economy: Exploring clothes swapping in emerging economies from a social practice perspective. Sustainable Development.

[B9-behavsci-15-00413] Chi T. (2015). Consumer perceived value of environmentally friendly apparel: An empirical study of Chinese consumers. The Journal of The Textile Institute.

[B10-behavsci-15-00413] D’Adamo I., Lupi G., Morone P., Settembre-Blundo D. (2022). Towards the circular economy in the fashion industry: The second-hand market as a best practice of sustainable responsibility for businesses and consumers. Environmental Science and Pollution Research.

[B11-behavsci-15-00413] De Keyser A., Lemon K. N., Klaus P., Keiningham T. L. (2015). A framework for understanding and managing the customer experience. Marketing Science Institute Working Paper Series.

[B12-behavsci-15-00413] Dragolea L. L., Butnaru G. I., Kot S., Zamfir C. G., Nuţă A. C., Nuţă F. M., Cristea D. S., Ştefănică M. (2023). Determining factors in shaping the sustainable behavior of the Generation Z consumer. Frontiers in Environmental Science.

[B13-behavsci-15-00413] Ek Styvén M., Mariani M. M. (2020). Understanding the intention to buy secondhand clothing on sharing economy platforms: The influence of sustainability, distance from the consumption system, and economic motivations. Psychology & Marketing.

[B14-behavsci-15-00413] Estévez A., Jauregui P., Granero R., Munguía L., López-González H., Macía L., López N., Momeñe J., Corral S., Fernández-Aranda F., Agüera Z., Mena-Moreno T., Lozano-Madrid M. d. E., Vintró-Alcaraz C., del Pino-Gutierrez A., Codina E., Valenciano-Mendoza E., Gómez-Peña M., Moragas L., Jiménez-Murcia S. (2020). Buying-shopping disorder, emotion dysregulation, coping and materialism: A comparative approach with gambling patients and young people and adolescents. International Journal of Psychiatry in Clinical Practice.

[B15-behavsci-15-00413] Evans F., Grimmer L., Grimmer M. (2022). Consumer orientations of secondhand fashion shoppers: The role of shopping frequency and store type. Journal of Retailing and Consumer Services.

[B16-behavsci-15-00413] Good M. C., Hyman M. R. (2020). Protection motivation theory and brick-and-mortar salespeople. International Journal of Retail & Distribution Management.

[B17-behavsci-15-00413] Gray S., Druckman A., Sadhukhan J., James K. (2022). Reducing the environmental impact of clothing: An exploration of the potential of alternative business models. Sustainability.

[B18-behavsci-15-00413] Huang H., Long R., Chen H., Sun K., Li Q. (2022). Exploring public attention about green consumption on Sina Weibo: Using text mining and deep learning. Sustainable Production and Consumption.

[B19-behavsci-15-00413] Hur E. (2020). Rebirth fashion: Secondhand clothing consumption values and perceived risks. Journal of Cleaner Production.

[B20-behavsci-15-00413] Iravanian A., Ravari S. O. (2020). Types of contamination in landfills and effects on the environment: A review study. IOP Conference Series: Earth and Environmental Science.

[B21-behavsci-15-00413] Jain V., O’Brien W., Gloria T. P. (2021). Improved solutions for shared value creation and maximization from used clothes: Streamlined structure of clothing consumption system and a framework of closed loop hybrid business model. Cleaner and Responsible Consumption.

[B22-behavsci-15-00413] Kacprzak A., Dziewanowska K. (2019). Investigating the influence of consumer socio-demographic characteristics on the preferred type of consumption experience. Journal of East European Management Studies.

[B23-behavsci-15-00413] Karpova E. E., Jestratijevic I., Lee J., Wu J. (2022). An ethnographic study of collaborative fashion consumption: The case of temporary clothing swapping. Sustainability.

[B24-behavsci-15-00413] Kawaf F., Tagg S. (2017). The construction of online shopping experience: A repertory grid approach. Computers in Human Behavior.

[B25-behavsci-15-00413] Khandual A., Pradhan S. (2019). Fashion brands and consumers approach towards sustainable fashion. Fast fashion, fashion brands and sustainable consumption.

[B26-behavsci-15-00413] King J., Wheeler A. (2016). Setting the record straight.

[B27-behavsci-15-00413] Kuuru T. K., Litovuo L., Aarikka-Stenroos L., Helander N. (2020). Emotions in customer experience. Society as an interaction space: A systemic approach.

[B28-behavsci-15-00413] Lang C., Zhang R. (2019). Second-hand clothing acquisition: The motivations and barriers to clothing swaps for Chinese consumers. Sustainable Production and Consumption.

[B29-behavsci-15-00413] Li S., Rismanchi B., Aye L. (2022). A simulation-based bottom-up approach for analysing the evolution of residential buildings’ material stocks and environmental impacts—A case study of Inner Melbourne. Applied Energy.

[B30-behavsci-15-00413] Liang D., Hou C., Jo M. S., Sarigöllü E. (2019). Pollution avoidance and green purchase: The role of moral emotions. Journal of Cleaner Production.

[B31-behavsci-15-00413] Lichy J., Ryding D., Rudawska E., Vignali G. (2023). Resale as sustainable social innovation: Understanding shifts in consumer decision-making and shopping orientations for high-end secondhand clothing. Social Enterprise Journal.

[B32-behavsci-15-00413] Long T., Suomi R. (2022). User continuance intention toward theme park apps: A uses and gratification perspective. 26th Pacific Asia Conference on Information Systems, PACIS 2022.

[B33-behavsci-15-00413] Malelak M. I., Anastasia N. (2020). Shopping motives, financial literacy, and credit card utilization among college students. Proceedings of the 3rd Asia Pacific international conference of management and business science (AICMBS 2019).

[B34-behavsci-15-00413] Mateos-Mínguez P., Arranz-López A., Soria-Lara J. A., Lanzendorf M. (2021). E-shoppers and multimodal accessibility to in-store retail: An analysis of spatial and social effects. Journal of Transport Geography.

[B35-behavsci-15-00413] McHugh M. L. (2013). The chi-square test of independence. Biochemia Medica.

[B36-behavsci-15-00413] Moes A., van Vliet H. (2017). The online appeal of the physical shop: How a physical store can benefit from a virtual representation. Heliyon.

[B37-behavsci-15-00413] Munsch A. (2021). Millennial and generation Z digital marketing communication and advertising effectiveness: A qualitative exploration. Journal of Global Scholars of Marketing Science.

[B38-behavsci-15-00413] Mustafy T., Rahman M. T. U. (2024). SPSS. Statistics and data analysis for engineers and scientists.

[B39-behavsci-15-00413] Nica E., Sabie O. M., Mascu S., Luţan A. G. (2022). Artificial intelligence decision-making in shopping patterns: Consumer values, cognition, and attitudes. Economics, Management and Financial Markets.

[B40-behavsci-15-00413] Ortega-Egea J. M., García-de-Frutos N., Antolín-López R. (2014). Why do some people do “more” to mitigate climate change than others? Exploring heterogeneity in psycho-social associations. PLoS ONE.

[B41-behavsci-15-00413] Park H., Martinez C. M. J. (2020). Secondhand clothing sales are booming—And may help solve the sustainability crisis in the fashion industry. The Conversation.

[B42-behavsci-15-00413] Park H. J., Lin L. M. (2020). Exploring attitude–behavior gap in sustainable consumption: Comparison of recycled and upcycled fashion products. Journal of Business Research.

[B43-behavsci-15-00413] Persson O., Hinton J. B. (2023). Second-hand clothing markets and a just circular economy? Exploring the role of business forms and profit. Journal of Cleaner Production.

[B44-behavsci-15-00413] Pramestya N. L. P. U. M., Widagda I. J. A. (2020). The role of positive emotion mediates fashion involvement on impulse buying. American Journal of Humanities and Social Sciences Research.

[B45-behavsci-15-00413] PwC (2018). Report Millennials vs Generation Z.

[B46-behavsci-15-00413] Rahadhini M. D., Wibowo E., Lukiyanto K. (2020). The role of positive emotion in hedonic shopping value affecting consumers’ impulse buying of fashion products. International Journal of Scientific and Technology Research.

[B47-behavsci-15-00413] Rapp A., Baker T. L., Bachrach D. G., Ogilvie J., Beitelspacher L. S. (2015). Perceived customer showrooming behavior and the effect on retail salesperson self-efficacy and performance. Journal of Retailing.

[B48-behavsci-15-00413] Reis C. L. D. S. (2020). Second-hand fashion: The effect of nostalgia on purchase intentions and shopping behaviour. Doctoral dissertation.

[B49-behavsci-15-00413] Rodrigues M., Proença J. F., Macedo R. (2023). Determinants of the purchase of secondhand products: An approach by the theory of planned behaviour. Sustainability.

[B50-behavsci-15-00413] Sachdeva I., Goel S. (2015). Retail store environment and customer experience: A paradigm. Journal of Fashion Marketing and Management.

[B51-behavsci-15-00413] Sendow F. F., Pangemanan S. S., Tielung M. V. (2019). Analysis of clicks and bricks: Consumers’ attitude towards online store and traditional store of Charles & Keith consumer in Manado. Jurnal EMBA: Jurnal Riset Ekonomi, Manajemen, Bisnis dan Akuntansi.

[B52-behavsci-15-00413] Simanjuntak M., Nur H. R., Sartono B., Sabri M. F. (2020). A general structural equation model of the emotions and repurchase intention in modern retail. Management Science Letters.

[B53-behavsci-15-00413] Sorensen K., Johnson Jorgensen J. (2019). Millennial perceptions of fast fashion and second-hand clothing: An exploration of clothing preferences using Q methodology. Social Sciences.

[B54-behavsci-15-00413] Štefancová V., Kalašová A., Čulík K., Mazanec J., Vojtek M., Mašek J. (2022). Research on the impact of COVID-19 on micromobility using statistical methods. Applied Sciences.

[B55-behavsci-15-00413] Tan C. S., Ooi H. Y., Goh Y. N. (2017). A moral extension of the theory of planned behavior to predict consumers’ purchase intention for energy-efficient household appliances in Malaysia. Energy Policy.

[B56-behavsci-15-00413] Tan T. M., Makkonen H., Kaur P., Salo J. (2022). How do ethical consumers utilize sharing economy platforms as part of their sustainable resale behavior? The role of consumers’ green consumption values. Technological Forecasting and Social Change.

[B57-behavsci-15-00413] Tarka P., Kukar-Kinney M., Harnish R. J. (2022). Consumers’ personality and compulsive buying behavior: The role of hedonistic shopping experiences and gender in mediating-moderating relationships. Journal of Retailing and Consumer Services.

[B58-behavsci-15-00413] Thangavel P., Pathak P., Chandra B. (2021). Millennials and Generation Z: A generational cohort analysis of Indian consumers. Benchmarking: An International Journal.

[B59-behavsci-15-00413] Trevinal A. M., Stenger T. (2014). Toward a conceptualization of the online shopping experience. Journal of Retailing and Consumer Services.

[B60-behavsci-15-00413] Turunen L. L. M., Cervellon M. C., Carey L. D. (2020). Selling second-hand luxury: Empowerment and enactment of social roles. Journal of Business Research.

[B61-behavsci-15-00413] Van der Horst M. (2024). Gender role attitudes. Encyclopedia of quality of life and well-being research.

[B62-behavsci-15-00413] Witek L., Kuźniar W. (2020). Green purchase behavior: The effectiveness of sociodemographic variables for explaining green purchases in emerging market. Sustainability.

[B63-behavsci-15-00413] Xu X. (2020). Examining the role of emotion in online consumer reviews of various attributes in the surprise box shopping model. Decision Support Systems.

[B64-behavsci-15-00413] Yan R. N., Bae S. Y., Xu H. (2015). Second hand clothing—Shopping among college students: The role of psychographic characteristics. Young Consumers.

[B65-behavsci-15-00413] Zhang X., Dong F. (2020). Why do consumers make green purchase decisions? Insights from a systematic review. International Journal of Environmental Research and Public Health.

[B66-behavsci-15-00413] Zulauf K., Wagner R. (2022). Online shopping therapy: If you want to be happy, shop around. Journal of International Consumer Marketing.

